# Cryopreservation without vitrification suitable for large scale cryopreservation of orchid seeds

**DOI:** 10.1186/s40529-018-0229-7

**Published:** 2018-05-09

**Authors:** Emily Schofield, Edward P. Jones, Viswambharan Sarasan

**Affiliations:** Natural Capital and Plant Health, Royal Botanic Gardens, Kew, Richmond, TW9 3AE UK

**Keywords:** Epiphyte, Ex situ conservation, Endemic, Madagascar, Lithophyte, Orchid seed, Terrestrial

## Abstract

**Background:**

Orchids are under threat from human activities and climate change, with populations limited to small geographic hotspots. This makes them ideal candidates for ex situ conservation. Orchid seeds are desiccation tolerant, but often have poor longevity in seed banks and cryopreservation of orchid protocorms is complex and expensive. Therefore, simple methods for large-scale storage programs are essential to store orchid seeds of different life forms. Seeds of five species representing epiphytic, lithophytic and terrestrial orchids from the Central Highlands of Madagascar were studied to find a simple and effective system of cryopreservation. The use of a vitrification solution prior to cryopreservation to improve survival was investigated, as well as the use of symbiotic and asymbiotic germination media to maximise germination after cryopreservation. Using the filter paper packet method, dried seeds were stored in vapour phase above liquid nitrogen and recovered after thawing with both symbiotic and asymbiotic media.

**Results:**

The study revealed that cryoprotection is not essential for the species in this study, which represented a range of lifeforms. Vitrification generally led to a decrease in germination post cryopreservation. The use of a symbiotic germination medium post cryopreservation was found to be successful in the species in which it was tested. However, the use of an asymbiotic medium was successful for all the species in this study.

**Conclusions:**

Vitrification was not essential for the species in this study as the orchid seeds were already ultralow temperature and desiccation tolerant. However, further studies using more species are required to validate this approach. This may be an ecophysiological or genetic trait of these species. Therefore, this form of dry seed cryopreservation could form part of ex situ orchid seed conservation using a standard method. The methods developed here will store greater genetic diversity compared to what can be achieved with protocorms and are suitable for both asymbiotic and symbiotic recovery after cryopreservation. This will help reduce the time and cost of ex situ conservation, and help develop universal protocols for large genera, compared to custom protocols required for protocorm cryopreservation.

**Electronic supplementary material:**

The online version of this article (10.1186/s40529-018-0229-7) contains supplementary material, which is available to authorized users.

## Background

The Orchidaceae is probably the largest family of flowering plants with more than 27,000 species (Zotz 2013) and is the most diverse plant group (Swarts and Dixon 2009), with high levels of ecological specialisation for pollinators and mycorrhizal fungi (Bailarote et al. 2012). The Itremo region in the Central Highlands of Madagascar is a micro-hotspot for orchids (Yokoya et al. 2015). The taxa we studied were from granite and quartz outcrops, and montane grassland. Seed banking is one of the cornerstones of ex situ biodiversity conservation. The majority of epiphytic orchids are desiccation tolerant, and can be stored at − 20 °C in seed banks (Seaton et al. 2010). According to Merritt et al. (2014) seed life span depends on seed traits, along with the biotic and abiotic components of their environment. Desiccation tolerance is common in most orchids, but longevity in storage using conventional seed banking is poor (Li and Pritchard 2009). Therefore, cryopreservation offers a viable long-term seed storage option (Popova et al. 2016).

Seed and protocorm cryopreservation utilises a range of different available methods including: direct freezing, encapsulation-vitrification and droplet-freezing, each tested in several taxa (Sakai and Engelmann 2007; Sopalun et al. 2010; Jitsopakul et al. 2012; Hu et al. 2013; Galdiano et al. 2014; Teixeira da Silva et al. 2014; Popova et al. 2016). However, protocorms, a widely used propagule for cryopreservation, are prone to cryopreservation injury due to their cellular structure and high-water content. Our preliminary studies using protocorms of selected species yielded no successful recovery (unpublished data). Use of seeds reduces the intrinsic risk of intracellular damage due to ice crystal formation (Kulus and Zalewska 2014) owing to low water content of mature seeds (Jitsopakul et al. 2008). Thousands of seeds can be stored in a relatively small space with low maintenance requirements (Popova et al. 2003). There is also the added benefit of storing a wider genetic diversity compared to what can be achieved using protocorms. This can be achieved either by storage in liquid nitrogen at − 196 °C or in its vapour phase around − 150 °C (Vendrame et al. 2014). Therefore, we are exploring the feasibility of dry seed cryopreservation without the use of a cryoprotectant and comparing symbiotic and asymbiotic media for post-cryopreservation recovery.

## Methods

The current study examines the viability of dry seed as propagules for the cryopreservation. To test this, three *Angraecum* species (*A. protensum*, *A. rutenbergianum* and *A. sesquipedale*) were cryopreserved with and without cryoprotectant. Vitrification using plant vitrification solution 2 (PVS2) was used in this study, due to its ability to remove and replace a large proportion of intracellular water from plant tissue (Kulus and Zalewska 2014). In addition, to test the use of symbiotic recovery after cryopreservation, *Angraecum magdalenae* and *Benthamia cinnabarina* were recovered using both asymbiotic and symbiotic methods. Popova et al. (2016) reviewed published seed cryopreservation to date and cryopreservation of both orchid and its fungal symbiont has been reported before as an experimental model (Batty et al. 2001; Teixeira da Silva et al. 2014). Merritt et al. (2014) proposed more advanced cryopreservation methods than currently available and simultaneous storage of seeds and fungal symbionts in a single system as potential area for future development. Our study takes the first step towards this by exploring possible simplification of the method of dry orchid seed cryopreservation. Therefore, our primary question concerns the development of a cost-effective and simple model to cryopreserve seeds from different life forms of orchids using dry seeds. Results will be discussed in the context of large scale ex situ programmes for threatened orchids from habitats where the threat to biodiversity is greater than anywhere else in the world.

## Plant species

Five taxa were chosen for this study representing epiphytic, lithophytic and terrestrial life forms (Table 1). Lithophytic and terrestrial groups require niche habitats and are less abundant in Madagascar compared to epiphytic taxa. The genus *Angraecum* consists of 210 species, of which 128 are endemic to Madagascar (Cribb and Hermans 2009). The research permits from the Madagascar Forestry Authority and CITES (Convention of International Trade in Endangered Species of Wild Flora and Fauna) permit for collecting the seeds are included as Additional file 1. We selected two lithophytic *Angraecum* species; *A. protensum* and *A. magdalenae* from quartz and granite outcrops, both of which grow on boulders or large rocks. Two epiphytic *Angraecum* species were collected, *A. rutenbergianum* from the Central Highlands of Madagascar (CHM) gallery forest and *A. sesquipedale* from a glasshouse collection in the Royal Botanic Gardens, Kew to investigate whether the observed desiccation tolerance traits were genetic or ecophysiological. The terrestrial species tested was *Benthamia cinnabarina,* a montane grassland species found in sandy soil at the borders of granite and quartz boulders (Table 1). Due to restrictions on collecting, a maximum of three capsules were collected from the wild, however, in many cases only one or two capsules were available for collecting at each site. The seeds used for each species were a mixture of seeds from at least 3 capsules. They were mixed before being stored at 4 °C.

**Table 1:** Orchid taxa and orchid mycorrhizal fungi studied, their life form and habitat in the wild

Orchid	Orchid life form	Habitat
*Angraecum magdalenae*	Lithophyte	On or near quartz boulders
*Angraecum protensum*	Lithophyte	Granite and quartz outcrops
*Angraecum sesquipedale*	Epiphyte	Sloping tree trunks in humid forest (collected from glasshouse at Royal Botanic Gardens Kew)
*Angraecum rutenbergianum*	Epiphyte	Tree trunks of evergreen humid forests
*Benthamia cinnabarina*	Terrestrial	Montane grassland in sandy soil at the borders of granite and quartz boulders

OTU (operational taxonomic unit) name of the mycorrhizal fungus (OMF)	Orchid life form of OMF partner	Habitat
Tul1	*Angraecum magdalenae* (Lithophyte)	Montane grassland in a crack in granite
Tul2	*Benthamia cinnabarina* (Terrestrial)	Montane grassland in sandy soil at the borders of granite and quartz boulders

### Orchid mycorrhizal fungi (OMF)

The different OMF (orchid mycorrhizal fungi) isolates (Tul denotes *Tulasnella*) Tul1 and Tul2 (Table 1) were collected from the Central Highlands of Madagascar. The isolates used in this study were collected, isolated and cultured as described by Yokoya et al. (2015) and isolates were selected from in vitro cultures stored at 18 °C.

### Seed sowing

Freshly collected mature seeds were cleaned and dried over LiCl for 2 weeks and stored at 4 °C until used for the study (approximately 1 year). Lithium chloride-mediated drying is a commonly used method (Galdiano et al. 2014). A pinch of seeds (ranging from 300 to 2000 seeds depending on the species) were transferred to hardened filter paper (Whatman, UK) and stapled. Due to the rare nature of the orchids and therefore limited seeds available, only one replicate per treatment was possible. Seed packets destined for direct sowing were surface sterilised in Magenta™ jars containing 50 ml of 0.5% sodium dichloroisocyanurate for 1 h. The other seeds were treated with or without vitrification solution. The vitrified seeds were then recovered and sterilised as described above. Once sterilised all seeds were washed in sterile water for 4 min and sown onto recovery medium 1 (RM1), Phytamax (Sigma, UK) with 3% sucrose and 0.15% activated charcoal, and recovery medium 2 (RM2), Oat Meal Agar medium (Clements 1988). RM2 was used for both asymbiotic and symbiotic recovery. The symbiotic RM2 consists of either OMF isolates Tul1 and Tul2 and (Rafter et al. 2016) (Table 1). Tul1 (originated from a lithophyte) and Tul2 (originated from a terrestrial) were specifically selected as these fungi were isolated from the mature roots of *Angraecum magdalenae* and *Benthamia cinnabarina* respectively, two of the taxa studied here (Table 1). Yokoya et al. (2015) were unsuccessful to isolate OMF from roots of both *A. protensum* and *A. sesquipedale* roots, therefore, only asymbiotic (RM1) medium was used for these species. Packets were cut open and seeds spread onto both asymbiotic (RM1) and symbiotic media (RM2). Non-cryopreserved controls were treated with PVS2 and then sown as above.

### Cryopreservation

#### Effect of vitrification on recovery of seeds after cryopreservation

Seeds, in seed packets as described above but without the sterilisation step, were treated directly in PVS2 for different times (0, 30, 45 and 60 min), and then transferred into Nunc™ cryovials (Sigma-Aldrich, Gillingham, UK) and covered with PVS2. Seeds with 0 min treatment were not treated with PVS2. Lids were put on both sets of cryovials, with and without PVS2 and dipped into liquid nitrogen on multi-vial trays, then put directly in the vapour phase above Liquid Nitrogen (LN) (− 170 ± 5 °C) in a D4000 Cryo Dewar (International Cryogenics, Indiana, USA) and stored for 1 week.

### Recovery

Cryovials containing seeds were removed from the Dewar and put in a water bath at 38 °C for 2 min. PVS2 solution was removed from the vial using Pasteur pipette and replaced with 1 ml of 1.2 M sucrose solution to completely cover the packet and then repeated twice. The 0 min PVS2 control vial was transferred directly into water bath for 2 min for thawing without sucrose solution. Seeds were sterilised after thawing, before sowing onto media as described above.

#### PVS toxicity

After the initial cryopreservation experiments *Angraecum protensum* seeds treated with plant vitrification solution 2 (PVS2) achieved very little germination. Therefore, the effect of vitrification was tested using Plant Vitrification Solutions PVS2 (30% (v/v) glycerol (Merck, Hertfordshire, UK), 15% (v/v) ethylene glycol (Sigma-Aldrich, Gillingham, UK), 15% (v/v) Dimethyl sulfoxide (DMSO) (Sigma-Aldrich, Gillingham, UK) and 0.4 M sucrose ½ Murashige and Skoog solution (½ MS) (Duchefa Biochemie, Haarlem, the Netherlands)) (Sakai et al. 1990) and PVS3 (50% glycerol, 50% sucrose (Sigma-Aldrich, Gillingham, UK)) (Nishizawa et al. 1993). *A. protensum* seeds were cryopreserved with either 30 or 60 min PVS2, PVS3 or no PVS.

### Data analysis

Germination was assessed 12 weeks after thawing as described by Yamazaki and Miyoshi (2006). The process of seed germination was divided into six categories according to the developmental stage of the embryos, adapted from Miyoshi and Mii (1995), as follows; Stage 0—cultured seed; Stage 1—imbibed seed; Stage 2—testa broken by emerging protocorm Stage 3—protocorm has emerged from the testa; Stage 4—protocorms with rhizoids; Stage 5—shoot primordium visible. Germination was determined to have occurred at stage 4 and 5 (protocorm and seedling stage). Assessments of germination were done under a stereo-microscope (Leica, Germany). Cryopreservation data was analysed using either an ANOVA or Two Sample T test in Microsoft Excel (Microsoft, Reading, UK). The effect of PVS2 and the recovery media used was investigated in cryopreserved and non-cryopreserved seeds.

## Results

### Effect of vitrification solutions on recovery of seeds after cryopreservation

**Fig. 1:**
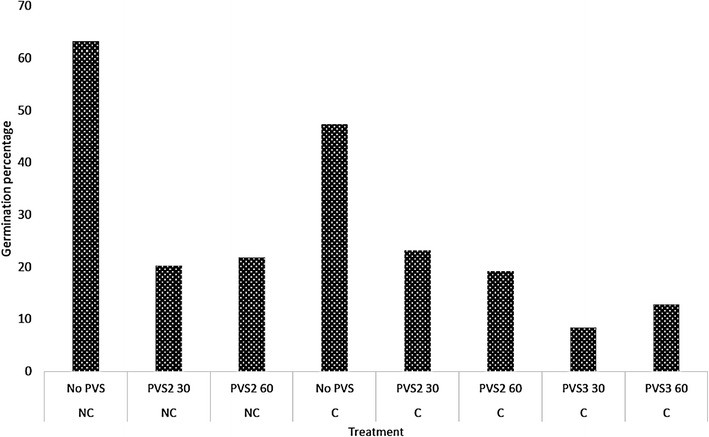
The effect of pre-treatment of PVS2 and PVS3 (30 and 60 min) on germination (protocorms and seedlings) of cryopreserved Angraecum protensum on recovery medium 1 (RM1) containing Phytamax nutrients. *C* cryopreserved, *NC* non-cryopreserved. Total viable seed tested = 10,535

In order to test the effect of vitrification in *Angraecum*, *A. protensum* was tested for PVS2 toxicity. Germination (protocorm and seedling stage combined) after PVS2 and PVS3 treatments was recorded to assess seed viability after vitrification and cryopreservation. The highest germination percentage after cryopreservation occurred when the seeds were not vitrified before cryopreservation (Fig. 1).

### Effect of vitrification on non-cryopreserved seeds

**Fig. 2:**
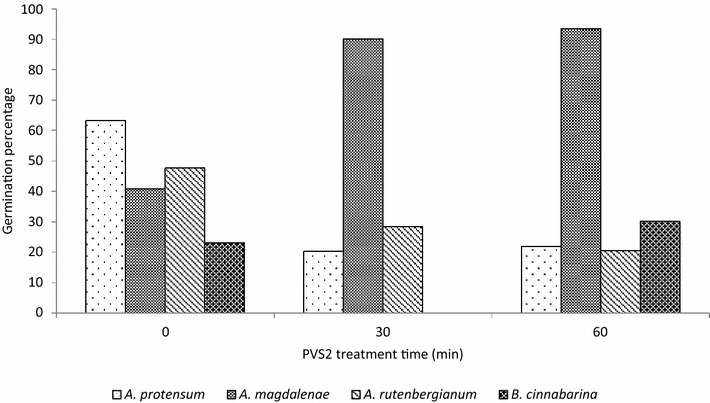
Percentage of germination (protocorms and seedlings) of non-cryopreserved seeds of four species of orchids (*Angraecum magdalenae*, *A. protensum*, *Benthamia cinnabarina* and *A. rutenbergianum*) treated with PVS2 and recovered on recovery medium 1 (RM1) containing Phytamax nutrients. Total viable seed tested = 3084

PVS2 reduced the average seed germination of all the tested species apart from *Angraecum magdalenae* when recovered with recovery medium 1 (RM1) without undergoing cryopreservation (Fig. 2). However, when the mean germination of all the tested species combined was analysed using an ANOVA test there was no significant difference between the average germination of seeds treated with or without PVS2 (F_2_ = 0.004, P = 0.99).

### Effect of vitrification on cryopreservation of seeds

**Fig. 3:**
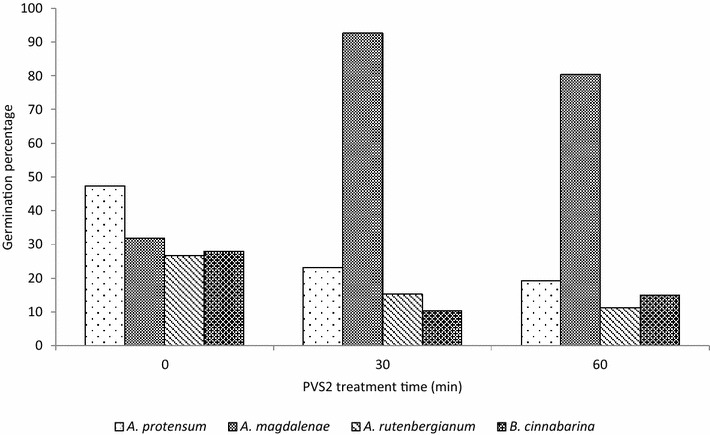
Germination (protocorms and seedlings) percentage of cryopreserved seeds of four species of orchids (Angraecum magdalenae, *A. protensum*, *A. rutenbergianum* and *Benthamia cinnabarina*) treated with PVS2 and recovered on recovery medium 1 containing Phytamax nutrients. Total viable seed = 3082

A similar pattern as that of the non-cryopreserved seed was found when seeds were treated with PVS2 and cryopreserved. The only species to have increased germination in response to cryopreservation following PVS2 treatment was *A. magdalenae.* A decrease in average germination was found for *A. protensum*, *A. magdalenae* and *A. rutenbergianum* (Fig. 2). There was also no significant difference in the mean germination across the four species when analysed using an ANOVA test (F_10_ = 0.02, P = 0.97) (Fig. 3).

## Traits of orchids from the same region

*Angraecum magdalenae* and *B. cinnabarina* occur in the same area of the Central Highlands of Madagascar. Therefore, these species were compared to test whether a requirement for PVS2 vitrification before cryopreservation is an ecophysiological or species-specific effect.

### *A. magdalenae*

**Fig. 4:**
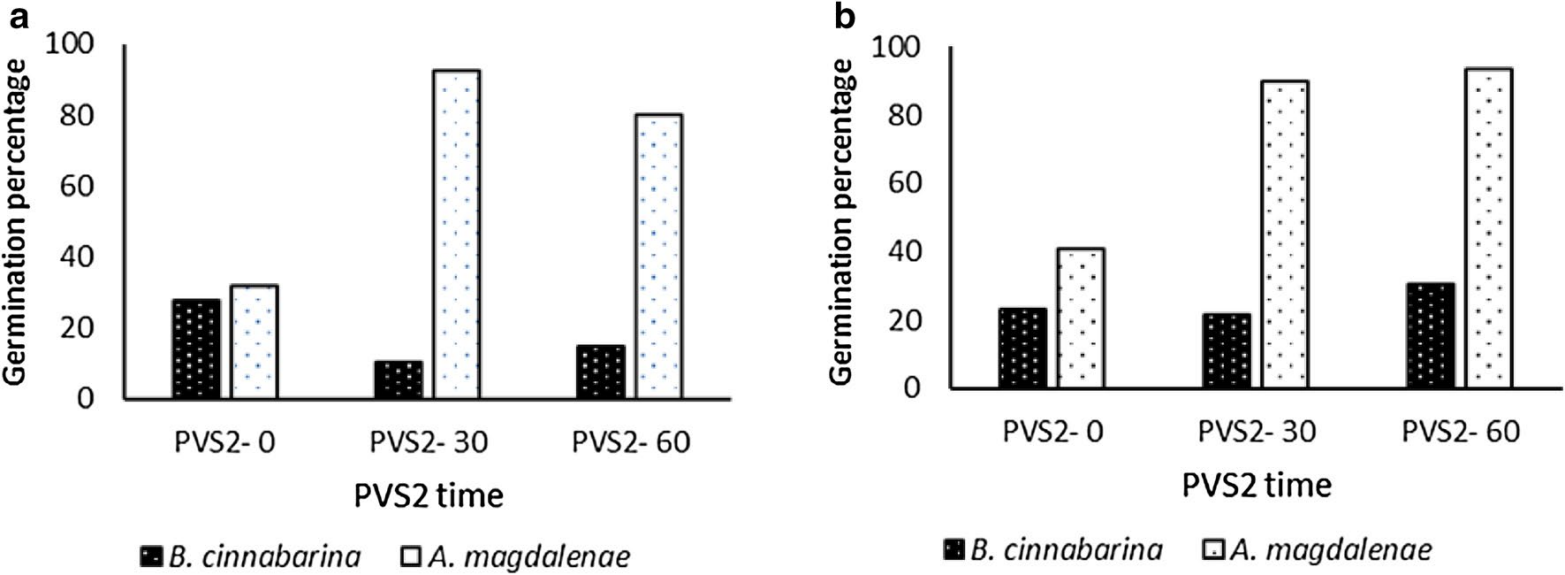
**a** Germination (protocorms and seedlings) percentage of *B. cinnabarina* and *A. magdalenae* cryopreserved with and without PVS2 treatment. Total seeds = 1969. **b** The germination percentage (protocorm and seedling) of non-cryopreserved *B. cinnabarina* and *A. magdalenae*. All seeds were recovered on recovery medium 1 (RM1) containing Phytamax nutrients. Total viable seeds tested = 1908

The lithophytic *A. magdalenae* behaved in a similar way to the terrestrial *B. cinnabarina*. Cryopreservation did not significantly reduce germination, compared to non-cryopreserved controls with 30 min PVS2 (χ_1_ = 0.0002, P = 0.98). However, unlike *B. cinnabarina*, *A. magdalenae* benefitted from PVS2 treatment (Fig. 4a). Significantly more recovery was found after cryopreservation with 30 min PVS2 compared to no PVS2 treatment (χ_1_ = 192.23, P = < 0.01).

### *B. cinnabarina*

Cryopreservation without vitrification was not harmful to seed recovery, with no significant difference in germination between the non-cryopreserved and cryopreserved treatments without PVS2 (χ_1_ = 0.34, *P *= 0.55), (Fig. 4b). Unlike *A*. *magdalenae,* PVS2 had no significant benefit to the recovery of cryopreserved *B. cinnabarina* seed, with no significant difference in recovery between 0 and 60 min PVS2 treatment (χ_1_ = 0.48, P= 0.48).

### Symbiotic vs. asymbiotic recovery

*Angraecum magdalenae* and *B. cinnabarina* were recovered symbiotically and asymbiotically to understand the relative benefits of symbiotic recovery in two species with different lifeforms (Table 2).

**Table 2:** Germination (protocorms and seedlings) of *Angraecum magdalenae* and *Benthamia cinnabarina* after cryopreservation with the optimum PVS2 treatment for each species

Medium	*A. magdalenae*	*B. cinnabarina*
Symbiotic (RM2 + fungus)	79.6	15.0
Asymbiotic (RM1)	92.5	23.2

RM2 tul1 was the symbiotic medium for *A. magdalenae* and RM2 tul2 for *B. cinnabarina*

### *Benthamiacinnabarina*

The symbiotic medium RM2 tul2 produced the maximum germination after 60 min PVS2 and cryopreservation, although this was not significantly more than the asymbiotic RM1 medium (χ_1_ = 1.04, P = 0.31). Both asymbiotic RM1 and symbiotic RM2 tul2 were found to facilitate germination to seedling stage.

### *Angraecummagdalenae*

No significant difference was found between recovery of cryopreserved seeds on the asymbiotic medium RM1 compared to the symbiotic medium RM2 tul1 (χ_1_ = 0.38, P = 0.54). Pre-treatment with 30 min PVS2 with recovery medium RM2 tul1 led to 65% germination, but only 13% progression to seedling stage. This contrasts with asymbiotic germination using RM1, which facilitated 66% of the germinated seeds to progress to seedling stage. Using a symbiotic recovery medium led to a high percentage of seeds becoming stuck at protocorm stage which could not progress to full seedling stage. This did not occur when the seeds were recovered on RM1.

## Discussion

### Need for vitrification before cryopreservation

This study clearly demonstrates that for the tested orchid seeds of *Angraecum* spp. and *B. cinnabarina* vitrification is not critical. Although vitrification is considered essential for the cryopreservation of protocorm and shoot tissue, direct cryopreservation of seeds without vitrification has been reported in other taxa (Popova et al. 2016). As more than one lifeform were tested, the lack of requirement for vitrification is unlikely to be due to a species or lifeform specific factor. This is in contrast to a number of previous studies of other tropical and subtropical species (Nikishina et al. 2001; Yamazaki and Miyoshi 2006), that are not traditionally considered desiccation and ultra-low temperature-tolerant (Popova et al. 2016). Orchid seeds are generally classified as orthodox with very low water content. However, further studies are required to determine the molecular mechanisms that allow cryopreservation without vitrification.

*Angraecum magdalenae* was the only species tested that significantly benefitted from vitrification before cryopreservation. Pre-treatment with 30 min PVS2 promoted germination to 92% compared to only 40% without PVS2 treatment. Despite the impressive germination after cryopreservation with PVS2, this is an exception to the general trend observed in this study. Previous studies have also successfully recovered *A. magdalenae* without vitrification (Nikishina et al. 2001). Therefore, we argue that the percentage of seeds successfully recovered without the use of PVS2 would be satisfactory for the long term cryogenic storage of *A. magdalenae*. If a less than optimum germination percentage could be accepted, then the need for expensive and time consuming extensive pre-screening of each individual orchid species before cryopreservation would be avoided.

*Benthamia cinnabarina* is from the granite and quart outcrops in the Central Highlands of Madagascar, like *A. protensum* and *A. magdalenae* studied here. As a terrestrial species we expected that *B. cinnabarina* would be more likely to require cryoprotection compared to the lithophytic species studied. However, *B. cinnabarina* did not require vitrification, whereas the lithophytic *A. magdalenae* benefitted from 30 min PVS2 pre-treatment. The mechanism behind the need for vitrification might be due to ecophysiological factors, as the terrestrial *B. cinnabarina* is adapted to a low moisture environment. However, *A. sesquipedale* seeds collected from the glasshouse at Kew also demonstrated desiccation and ultralow temperature tolerance, being cryopreserved most successfully without vitrification. This suggests that this response may also have a genetic basis. By conducting larger trials in different species of *Benthamia* and *Angraecum* from a range of habitat types will shed light on the traits that are linked to seed tolerance of ultralow temperature.

A universal cryopreservation method has yet to be found that is suitable for the seeds of a range of orchid species (Popova et al. 2016). Therefore, the observation that vitrification is not critical for cryopreservation in the studied *Angraecum* and *Benthamia* species from Madagascar has significance to the conservation of other related species from the genera and beyond. This finding could contribute to further studies in 128 species of *Angraecum* and more than 35 species of *Benthamia* for quick screening and development of generalised cryopreservation protocols. Habitat destruction continues at an unprecedented pace in the Central Highlands of Madagascar, leading to habitat fragmentation (Ramiadantsoa et al. 2015), and driving many endemic orchids to the verge of extinction.

### Symbiotic recovery of seeds

Asymbiotic recovery media is the current standard approach to recover cryopreserved orchid seeds. However, symbiotic germination of orchid seeds is more successful when reintroducing orchids into the wild (Johnson et al. 2007). Most mature orchids produce multiple seed pods each season, each containing thousands of seeds (Swarts and Dixon 2009). Therefore, 30–50% recovery after cryopreservation would still be able to potentially produce hundreds or even thousands of plants for re-establishment of orchid populations in the wild. Cryopreservation without vitrification will help develop low-cost cryopreservation methods, as described by Li and Pritchard (2009).

Maximum *A. magdalenae* germination was achieved with asymbiotic recovery. Despite the tul2 isolate being able to promote germination after cryopreservation, it was unable to promote germination to seedling stage. Phytamax was effective at germinating all the species in this study. It contains nitrogen in a form which is accessible to the plant, organic compounds and sucrose, all components required for the germination of many different orchid species (Dutra et al. 2008). The selected fungus used for symbiotic recovery in this study may have been unable to provide the appropriate nutrients or sugars to the germinating orchid seed. This highlights the vital importance of selecting the appropriate mycorrhizal fungus to promote germination to seedling stage, and the need for large scale screening of mycorrhizal fungal isolates to identify these for each cryopreserved orchid species.

Previous studies have either focussed on encapsulation of the seed and mycorrhizal fungus (Wood et al. 2000) or separate storage and recovery of the orchid and its symbiont (Sommerville et al. 2008; Ercole et al. 2013). These require initial investment into finding an appropriate mycorrhizal partner and optimisation of storage conditions for both the fungus and orchid. This is likely to be a secondary step and less suitable for orchid species that are under immediate threat, due to the time required for optimisation.

## Conclusions

This study across many orchid species has found that PVS2 is not a requirement for ex situ storage of orchid seeds from the Central Highlands of Madagascar. Despite *A. magdaleane* benefitting from PVS2 pre-treatment before cryopreservation, sufficient germination after cryopreservation without vitrification was achieved for potential reintroduction to take place. This approach could be the basis of a large scale ex situ conservation strategy for orchids under immediate threat of extinction. Aiming for sufficient instead of optimum germination is a good initial step to protect critically endangered orchids. This can then be followed up with a more specific symbiotic storage method using a compatible mycorrhizal fungus to aid the production of seedlings suitable for long term reintroduction to the wild.

## Additional file

**Additional file 1.** Raw data.

## Abbreviations

PVS2: plant vitrification solution 2
PVS3: plant vitrification solution 3
CITES: Convention on International Trade of Endangered Species of Wild Flora and Fauna
CHM: Central Highlands of Madagascar
OTU: operational taxonomic unit
OMF: orchid mycorrhizal fungi
RM1: recovery medium 1
RM2: recovery medium 2
Tul1: tulasnella isolate 1
Tul2: tulasnella isolate 2
